# P24 Antimicrobial stewardship in an outpatient home IV antibiotic therapy programme for chronic pulmonary infections: microbiology, prescribing patterns and opportunities for optimization

**DOI:** 10.1093/jacamr/dlaf230.031

**Published:** 2025-12-04

**Authors:** Milton James Micallef, Novis Kola, Lucy Carson, Uta Hill

**Affiliations:** Royal Papworth Hospital NHS Foundation Trust, Papworth Road, Cambridge Biomedical Campus, Cambridge, CB2 0AY, UK; UNSW Sydney, Prince of Wales Clinical School, Randwick, New South Wales 2031, Australia; Royal Papworth Hospital NHS Foundation Trust, Papworth Road, Cambridge Biomedical Campus, Cambridge, CB2 0AY, UK; Royal Papworth Hospital NHS Foundation Trust, Papworth Road, Cambridge Biomedical Campus, Cambridge, CB2 0AY, UK; Royal Papworth Hospital NHS Foundation Trust, Papworth Road, Cambridge Biomedical Campus, Cambridge, CB2 0AY, UK

## Abstract

**Objectives:**

To describe the microbiology and prescribing patterns in a tertiary home IV antibiotic therapy (OPAT) programme for chronic pulmonary infections, assess antimicrobial stewardship (AMS) practices and identify opportunities for optimization.

**Methods:**

A retrospective audit was conducted of all patients treated via our patient/carer-administered OPAT service between 1 January and 31 December, 2023, excluding those who opted out via the NHS National Data Opt-out service (*n*=26 patients, 39 courses excluded). Eligible patients included people with cystic fibrosis (pwCF) and those with non-cystic fibrosis-related (non-CF) chronic lung disease. Data collected included demographics, patient cohort, antibiotic regimen, treatment duration, sputum microbiology, C-reactive protein (CRP) change, adverse events and reasons for early cessation. Appropriateness of therapy was assessed against recent (within 12 months) or contemporary sputum culture results.

**Results:**

A total of 413 treatment episodes were delivered to 274 patients (74 pwCF courses, 339 non-CF courses). Sputum cultures were attempted in 374 courses (91%), yielding at least one isolate in 263 specimens, most commonly *Pseudomonas aeruginosa*. Only six courses lacked prior microbiology data within 12 months. There was no significant growth in 110 samples. Meropenem (*n*=188) and ceftazidime (*n*=107) were the most frequently prescribed agents; piperacillin/tazobactam was less frequently used (*n*=93) due to the 6 h dosing for *P. aeruginosa* deemed burdensome in the home setting. The mean treatment duration was 13·8 days (SD 4·4; range 2–32). Mean CRP fell from 14 mg/L (SD 24; range 3–200) to 11 mg/L (SD 20; range 3–219), a reduction of 4 mg/L. Adverse events were recorded in 25 courses leading to early cessation, including non-severe cutaneous (*n*=10), subjective (*n*=6), gastrointestinal (*n*=4), liver function derangements (*n*=3), hyponatraemia (*n*=1) and fluid overload (*n*=1). No line-related bloodstream infections occurred. Two patients tested positive for *Clostridioides difficile*, however, one of these was toxin-negative, and the other was positive 6 months after the course of home IV antibiotics.

**Conclusions:**

Home IV antibiotic treatment for complex respiratory infections, self or carer-administered in a tertiary ambulatory care setting is safe, with prescribing patterns influenced by pathogen profile, patients’ previously demonstrated intolerances, drug stability and dosing practicality. AMS opportunities include clearer documentation of diagnostic reasoning and antibiotic selection, closer integration with microbiology, and consideration of early-response-guided treatment duration (STOP-2 trial; forthcoming results from SBIVA trial underway). These strategies could further optimize OPAT effectiveness while preserving antimicrobial efficacy.

